# Developing an interpretable machine learning model via SHAP to predict HCC postoperative survival based on tumor immune microenvironment CODEX immunomics and MRI

**DOI:** 10.1186/s40644-026-01006-y

**Published:** 2026-02-14

**Authors:** Wenjie Zou, Kangsheng Peng, Muye Yang, Yingxi Zhang, Wanming Liu, Ningyang Jia, Kairong Song, Jiaping Xu, Peijun Wang

**Affiliations:** 1 Department of Radiology, Tongji Hospital, School of Medicine, Tongji University, Shanghai, China; 2 Department of Gastroenterology, Shanghai Tenth People’s Hospital, Tongji University School of Medicine, Shanghai, China; 3 Department of Pathology, Tongji Hospital, School of Medicine, Tongji University, Shanghai, China; 4 Department of Radiology, Ruijin Hospital, Shanghai Jiao Tong University School of Medicine, Shanghai, China; 5 Department of Radiology, Eastern Hepatobiliary Surgery Hospital, The Third Affiliated Hospital of Naval Medical University, Shanghai, China; 6 Department of Medical Imaging, Suzhou Medical College, Soochow University, Suzhou, China

**Keywords:** Hepatocellular carcinoma, CODEX immunomics, Machine learning, Tumor immune microenvironment, Survival prediction, SHAP

## Abstract

**Objective:**

By generating an immune score reflecting the tumor immune microenvironment via Co-detection by Indexing (CODEX) Immunomics and integrating clinicoradiological features, we developed an interpretable machine learning model to predict postoperative survival in hepatocellular carcinoma (HCC) using SHapley Additive exPlanations (SHAP).

**Methods:**

We retrospectively enrolled 94 HCC patients who underwent the CODEX procedure and had preoperative magnetic resonance imaging. Patients were divided into a training set (*n* = 65) and a validation set (*n* = 29) in a 7:3 ratio. Univariate and multivariate Cox regression analyses identified clinicoradiological independent risk factors for 5-year survival to construct the Clinical model. For immunomics analysis, 36 immune-related molecules were evaluated using CODEX. Key features were selected through univariate Cox regression and Recursive Feature Elimination (RFE). The best-performing classifier among five machine learning algorithms was used to build the Immune model. The immune score from the Immune model and variables from the Clinical model were combined using multivariate Cox regression to identify independent risk factors, forming the Clinical-Immune model. Models were compared for discrimination, calibration, and clinical utility. SHAP was used to interpret the model’s predictions.

**Result:**

Shape, arterial peritumoral enhancement, intratumoral necrosis constituted the Clinical model. Five immunomics features formed the Immune model using a survival decision algorithm. The Clinical-Immune model combined the immune score and arterial peritumoral enhancement. The concordance indexes (C-indexes) for the three models were 0.730, 0.832, and 0.852 in the training set, and 0.624, 0.815, and 0.870 in the validation set. Time-dependent area under the curve (timeAUC) values were 0.833, 0.907, and 0.969 in the training set, and 0.656, 0.919, and 1.000 in the validation set. The Clinical-Immune model, which demonstrated the best performance and offered superior predictive consistency and clinical utility, was selected as the final prediction model.

**Conclusion:**

We developed an interpretable machine learning model to predict postoperative survival in HCC patients using CODEX immunomics and clinicoradiological features. This robust model enhances survival prediction and supports clinical decision-making in HCC management.

**Supplementary Information:**

The online version contains supplementary material available at 10.1186/s40644-026-01006-y.

## Introduction

Hepatocellular carcinoma (HCC) is the most prevalent type of liver cancer and a significant global health issue, ranking as the third leading cause of cancer deaths and the sixth most diagnosed [1]. Despite advances in treatments like surgery, liver transplants, and systemic therapies, the 5-year survival rate for HCC remains below 50% [2–5]. Accurate prediction of postoperative survival is crucial for better treatment and patient outcomes. However, current prognostic models for HCC primarily rely on clinical, radiological, or pathological features [6–8].Radiological findings are increasingly incorporated into preoperative risk models and nomograms for abdominal malignancies, underscoring the prognostic value of imaging biomarkers [9]. These models often fail to capture the complex biological and molecular mechanisms underlying HCC progression [10, 11]. Tumor heterogeneity further complicates accurate prognosis. Traditional models lack the ability to integrate complex biological data, highlighting the need for approaches that incorporate multi-dimensional data to enhance predictive accuracy.

The tumor immune microenvironment (TME) plays a crucial role in cancer metastasis, progression, therapeutic response, and patient prognosis [12–14]. The TME in HCC is particularly complex, involving various immune cells and immune-related molecules. A comprehensive indicator, the immune score, is essential for TME assessment. However, previous immune score evaluations often relied on multiple immunohistochemistry sections or a limited subset of immune cells, resulting in an incomplete assessment of the TME [15]. Additionally, its assessment requires biopsies, potentially promoting tumor metastasis. To overcome these limitations, we have adopted the Co-detection by Indexing (CODEX) immunomics technology.

CODEX is a newly developed and commercially available technology by Akoya Biosciences that enables highly multiplexed tissue imaging at the single-cell level [16, 17]. Unlike methods such as multiplex immunohistochemistry, cyclic immunofluorescence, and Cell DIVE, CODEX applies all antibodies to the tissue sample simultaneously in a single staining procedure, reducing staining time and preserving epitopes [18]. It uses oligonucleotide-conjugated antibodies and sequential fluorescent reporters to detect up to 60 protein markers simultaneously in a single tissue, allowing for a comprehensive evaluation of the TME without invasive biopsies [16–18].

Current research on CODEX mainly focuses on mapping cellular biogeography [19], annotating cell types [20], and quantifying cellular arrangements [21]. However, no existing studies have used CODEX immunomics to quantify the TME and combine it with clinical and radiological factors to predict the survival of HCC patients. This study aims to fill this gap by applying CODEX immunomics to quantify the TME in HCC and integrating these data with clinical and radiological features to develop an interpretable machine learning model for predicting postoperative survival. This model significantly improves the accuracy of survival predictions and supports clinical decision-making, offering a novel approach for multidimensional prediction in liver cancer and potentially other cancers.

## Materials and methods

### Patients

**Fig. 1:**
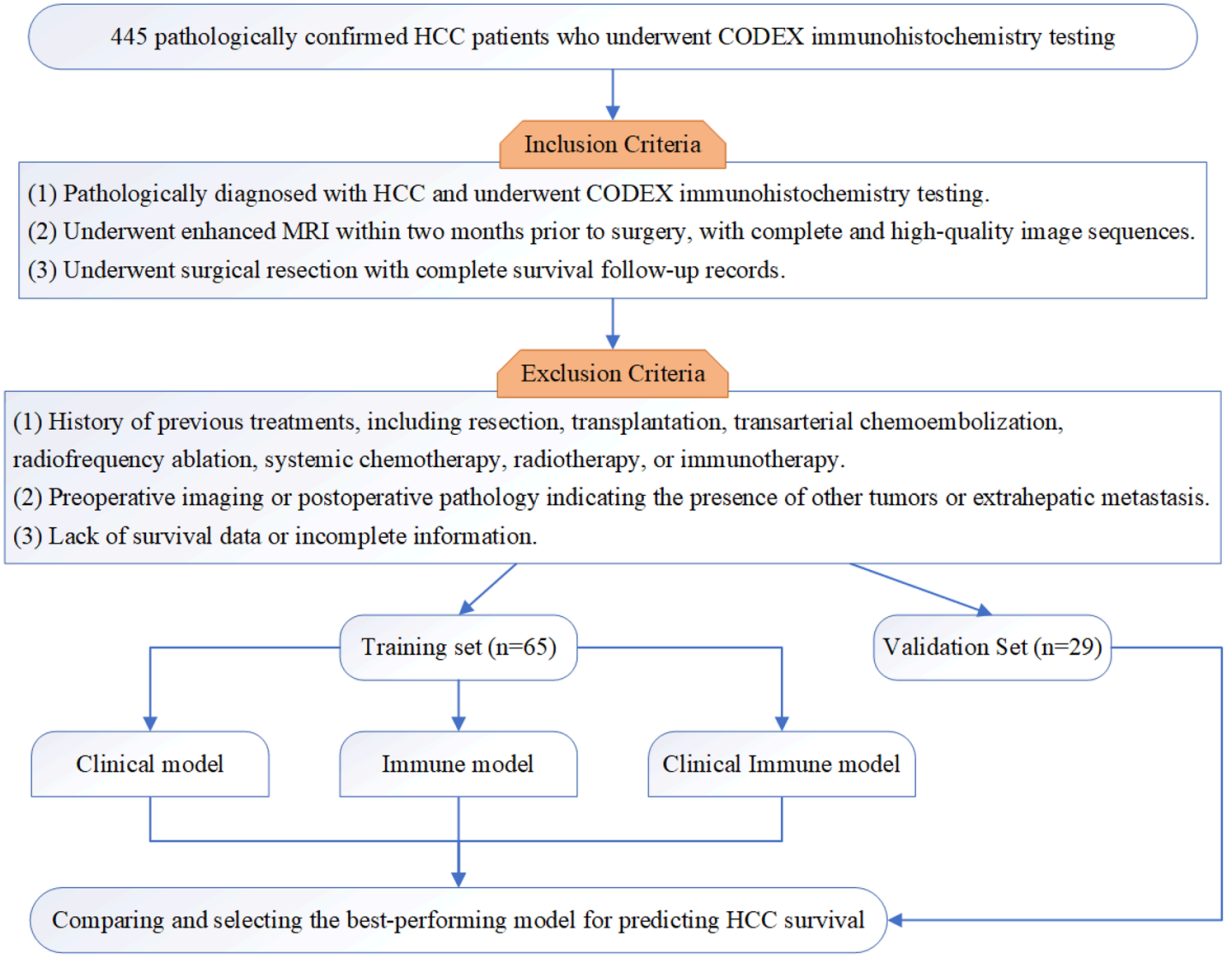
The flowchart of patient selection

This retrospective study included 445 pathologically confirmed HCC patients who underwent CODEX immunohistochemistry testing between February 2010 and December 2021. After applying the inclusion and exclusion criteria, 94 patients were selected and randomly divided into a training set (65 patients) and a validation set (29 patients) in a 7:3 ratio. The patient selection flowchart is shown in Fig. 1. This study was approved by the Ethics Committee of the Eastern Hepatobiliary Surgery Hospital, The Third Affiliated Hospital of Shanghai Naval Military Medical University (EHBHKY2018-1-001). According to national laws and institutional regulations, written informed consent was not required.

Inclusion criteria: (1) Pathologically diagnosed with HCC and underwent CODEX immunohistochemistry testing. (2) Underwent enhanced MRI within two months prior to surgery, with complete and high-quality image sequences. (3) Underwent surgical resection with complete survival follow-up records.

Exclusion criteria: (1) History of previous treatments, including resection, transplantation, transarterial chemoembolization, radiofrequency ablation, systemic chemotherapy, radiotherapy, or immunotherapy. (2) Preoperative imaging or postoperative pathology indicating the presence of other tumors or extrahepatic metastasis. (3) Lack of survival data or incomplete information.

### Clinical, laboratory, and survival data collection

Clinical and laboratory indicators were obtained from medical records and the laboratory information system, respectively. The collected indicators are listed in Table 1.

**Table 1:** Baseline characteristics in training and validation sets for predicting 5-Year survival

Characteristic	Training set (*n* = 65)			Validation set (*n* = 29)		
	survival (*n* = 32)	death (*n* = 33)	*P* value	survival (*n* = 14)	death (*n* = 15)	*P* value
**Clinical features**						
Osday	1886.5 (1825–2279)	440(187–1001)	**< 0.001**	1825(1786–1825)	545(251.5–873)	**< 0.001**
Age	59.00 (43.75-64)	58 (53–63)	0.482	53.93 ± 8.05	56.33 ± 12.73	0.552
Gender						
Male	29 (90.6%)	28 (84.8%)	0.741	13 (92.9%)	13 (86.7%)	1.000
Female	3 (9.4%)	5 (15.2%)		1 (7.1%)	2 (13.3%)	
AFP(g/L)	13.15 (5.42-269.85)	29.5(4.7-661.3)	0.748	65.65(6.52-363.04)	95.8(3.35-966.46)	0.827
CA199(U/mL)	17.70 (8.10-24.32)	26.7(14-48.92)	0.051	20.82 ± 12.12	19.67 ± 12.09	0.800
CEA(ng/mL)	2.35 (1.48–3.23)	3.00 (2.30–3.9)	0.053	2.46 ± 0.99	2.53 ± 1.17	0.862
ALT(U/L)	23.35 (19-39.8)	33(29.60–64)	**0.003**	29.50 (21.25-70)	35(27.5–42.3)	0.793
AST(U/L)	25(20.85–38.4)	32(26.2–90)	**0.012**	28.5(21.75–56.9)	37(29.85-62)	0.295
TP(g/L)	70.25 (65.25–74.3)	69.4(60.7–73.2)	0.201	66.79 ± 4.99	67.75 ± 4.70	0.599
ALB(g/L)	42.15(39.1-45.35)	40.7 (37.8–43.4)	0.179	40.15 (37.55–41.55)	41.6(38.65–43.5)	0.348
GLOB(g/L)	28.65 ± 4.73	27.82 ± 4.43	0.471	27.90 ± 3.71	26.92 ± 2.17	0.390
TBIL(µmol/L)	14.05(11.35–18.32)	11.5 (9.8–16.4)	0.203	15.65 (12.88–19.2)	12(8.6-15.35)	**0.045**
DBIL(µmol/L)	5.40 (3.98–7.75)	5.30 (4.30–7.2)	0.865	6.79 ± 2.82	4.73 ± 1.53	**0.020**
IBIL(µmol/L)	8.65 (7.07-10)	6.30 (5.80-9)	**0.021**	9.34 ± 2.98	7.19 ± 2.61	**0.048**
GGT(U/L)	57.5(31-84.25)	94(56–105)	**0.025**	61.5(38.25-197.25)	105(44.15–125)	0.678
AFU(U/L)	22(17.00-27.25)	21 (17–27)	0.969	25.49 ± 9.29	22.57 ± 6.58	0.336
PLT(10^9/L)	163(123-189.5)	158(124.00-237)	0.922	121.71 ± 54.06	150.47 ± 65.13	0.209
PT(S)	11.6(10.90-12.15)	12(11.2–12.7)	0.220	11.8(11.33–12.2)	11.9(11.25–12.35)	0.965
APTT(S)	26.47(25.68–28.18)	26.34 (25.88–27.66)	0.984	26.52 ± 3.25	26.59 ± 1.90	0.943
TT(S)	18.90 ± 1.22	18.70 ± 1.38	0.530	19.17(18.69–19.68)	18.3(17.7-19.12)	0.022
FBG(g/L)	2.50 (2.30–2.78)	2.52 (2.33–2.86)	0.778	2.32 ± 0.55	2.62 ± 0.58	0.160
CHOL(mmol/L)	4.19 (3.64–4.57)	4.24 (3.92–4.47)	0.600	4.06 (3.34–4.60)	4.13 (3.92–4.59)	0.646
TG(mmol/L)	1.21 (0.97–1.37)	1.14 (0.89–1.27)	0.309	0.98 (0.89–1.46)	1.13 (0.94–1.21)	0.965
HDL-C(mmol/L)	1.07 (1.00-1.19)	1.15 (1.02–1.21)	0.201	1.12 (1.05–1.26)	1.09 (1.02–1.15)	0.743
LDL-C(mmol/L)	2.47 (2.21–3.14)	2.79 (2.31–3.06)	0.609	2.72 ± 1.07	2.83 ± 0.50	0.728
HBsAg						
Negative	9 (28.1%)	13 (39.4%)	0.485	4 (28.6%)	6 (40.0%)	0.798
Positive	23 (71.9%)	20 (60.6%)		10 (71.4%)	9 (60.0%)	
HBsAb						
Negative	26 (81.2%)	20 (60.6%)	0.120	12 (85.7%)	11 (73.3%)	0.716
Positive	6 (18.8%)	13 (39.4%)		2 (14.3%)	4 (26.7%)	
HBeAg						
Negative	30 (93.8%)	31 (93.9%)	1.000	13 (92.9%)	12 (80.0%)	0.642
Positive	2 (6.2%)	2 (6.1%)		1 (7.1%)	3 (20.0%)	
HBeAb						
Negative	6 (18.8%)	13 (39.4%)	0.120	3 (21.4%)	5 (33.3%)	0.763
Positive	26 (81.2%)	20 (60.6%)		11 (78.6%)	10 (66.7%)	
HBcAb						
Negative	4 (12.5%)	3 (9.1%)	0.966	1 (7.1%)	1 (6.7%)	1.000
Positive	28 (87.5%)	30 (90.9%)		13 (92.9%)	14 (93.3%)	
**MRI features**						
Tumor diameter(cm)	5.05 (3.20–6.12)	6.80 (4.30–9.90)	0.069	4.05 (2.80–5.80)	7.90 (5.70–9.35)	0.063
Tumor number						
Solitary	28 (87.5%)	27 (81.8%)	0.370	14 (100.0%)	13 (86.7%)	0.367
Multiple	4 (12.5%)	6(18.2%)		0 (0.0%)	2 (13.4%)	
Shape						
Regular	24 (75.0%)	14 (42.4%)	**0.016**	9 (64.3%)	5 (33.3%)	0.195
Irregular	8 (25.0%)	19 (57.6%)		5 (35.7%)	10 (66.7%)	
Margin						
Smooth	15 (46.9%)	13 (39.4%)	0.720	10 (71.4%)	5 (33.3%)	0.093
Non-smooth	17 (53.1%)	20 (60.6%)		4 (28.6%)	10 (66.7%)	
Radiological capsule enhancement						
Complete	10 (31.2%)	10 (30.3%)	0.317	5 (35.7%)	6 (40.0%)	0.589
Incomplete	15 (46.9%)	20 (60.6%)		5 (35.7%)	7 (46.7%)	
Absent	7 (21.9%)	3 (9.1%)		4 (28.6%)	2 (13.3%)	
Nonrim APHE						
Present	20 (62.5%)	20 (60.6%)	1.000	9 (64.3%)	7 (46.7%)	0.562
Absent	12 (37.5%)	13 (39.4%)		5 (35.7%)	8 (53.3%)	
Rim APHE						
Absent	20 (62.5%)	21 (63.6%)	1.000	8 (57.1%)	6 (40.0%)	0.581
Present	12 (37.5%)	12 (36.4%)		6 (42.9%)	9 (60.0%)	
Arterial peritumoral enhancement						
Absent	28 (87.5%)	16 (48.5%)	**0.002**	11 (78.6%)	4 (26.7%)	**0.015**
Present	4 (12.5%)	17 (51.5%)		3 (21.4%)	11 (73.3%)	
Nonperipheral"washout”						
Present	19 (59.4%)	19 (57.6%)	1.000	9 (64.3%)	9 (60.0%)	1.000
Absent	13 (40.6%)	14 (42.4%)		5 (35.7%)	6 (40.0%)	
Enhancement pattern						
Typical	19 (59.4%)	17 (51.5%)	0.698	8 (57.1%)	7 (46.7%)	0.847
Atypical	13 (40.6%)	16 (48.5%)		6 (42.9%)	8 (53.3%)	
Intratumoral necrosis						
Absent	19 (59.4%)	7 (21.2%)	**0.004**	3 (21.4%)	6 (40.0%)	0.497
MRI liver cirrhosis						
Present	13 (40.6%)	26 (78.8%)		11 (78.6%)	9 (60.0%)	
Absent	15 (46.9%)	6 (18.2%)	**0.027**	7 (50.0%)	2 (13.3%)	0.083
Present	17 (53.1%)	27 (81.8%)		7 (50.0%)	13 (86.7%)	
Splenomegaly						
Absent	25 (78.1%)	20 (60.6%)	0.207	11 (78.6%)	14 (93.3%)	0.540
Present	7 (21.9%)	13 (39.4%)		3 (21.4%)	1 (6.7%)	
Ascites						
Absent	27 (84.4%)	19 (57.6%)	**0.036**	9 (64.3%)	10 (66.7%)	1.000
Present	5 (15.6%)	14 (42.4%)		5 (35.7%)	5 (33.3%)	

Abbreviations: Osday, overall survival days; AFP, alpha-fetoprotein; CA199, carbohydrate antigen 19 − 9; CEA, carcinoembryonic antigen; ALT, alanine aminotransferase; AST, aspartate aminotransaminase; TP, total protein; ALB, albumin; GLOB, globulin; TBIL, total bilirubin; DBIL, direct bilirubin; IBIL, indirect bilirubin; GGT, r-glutamyltransferase; AFU, a-fucosidase; PLT, platelet count; PT, prothrombin time; APTT, activated partial thromboplastin time; TT, thrombin time; FBG, fibrinogen; CHOL, total cholesterol; TG, triglyceride; HDL-C, high density lipoprotein cholesterol; LDL-C, low density lipoprotein cholesterol; APHE, arterial phase hyperenhancement

Patients were followed up starting from the first day after surgery, recorded in days. Within the first two years postoperatively, patients required an upper abdominal enhanced Computed Tomography or MRI scan and related laboratory tests (such as alpha-fetoprotein, etc.) every three months. Afterward, patients underwent regular follow-up every six months. The end date of the follow-up was set at five years postoperatively, which is 1825 days. Follow-up information of patients will be retrieved from the hospital’s medical record system and supplemented by telephone follow-up. The survival outcome variables for patients were whether they survived and the overall survival days (OSdays).

### MRI contrast-enhanced scan protocol

Patients underwent MR scanning using a 1.5T scanner and an 8-channel abdominal coil (Optima MR360, GE Healthcare). Patients underwent MR scanning using a 1.5T scanner and an 8-channel abdominal coil (Optima MR360, GE Healthcare). All patients fasted for at least 4–8 h and refrained from drinking water for at least 4 h before the scan. Gadopentetic acid (Gd-DTPA) or Gadobenate Dimeglumine (Gd-BOPTA) was injected into the antecubital vein using a high-pressure injector at a rate of 1.5-2.0 ml/s, with a total dose of 0.1 mmol/kg, followed by a 20 ml saline flush over approximately 20 s. Scans were performed at 20–35 s, 55–75 s, and 150–180 s after contrast agent injection, corresponding to the arterial phase (AP), portal venous phase (PVP), and delayed phase (DP), respectively. Additionally, MRI sequences included fat-suppressed T2-weighted imaging (T2WI), T1-weighted imaging (T1WI), diffusion-weighted imaging (DWI), and apparent diffusion coefficient (ADC) mapping. Detailed scan parameters are provided in Supplementary Table S1.

### MRI feature evaluation

Two radiologists with over 5 years of experience in abdominal MRI (LWM and SKR) independently analyzed the MR imaging features of all patients. They were blinded to all information except the pathological confirmation of HCC. After the analysis, interobserver agreement for all imaging features was assessed using Kappa analysis. Features with a Kappa value less than 0.85 were excluded. For features with discrepancies, a third radiologist with over 10 years of experience in liver MRI (NYJ) was invited to participate in a discussion and make the final decision. The consensus decisions of the three radiologists were used for subsequent statistical analysis.

Fifteen imaging features were evaluated: (a) Tumor diameter; (b) Tumor number; (c) Shape; (d) Margin; (e) Radiological capsule enhancement; (f) Nonrim Arterial Phase Hyperenhancement (Nonrim APHE); (g) Rim APHE; (h) Nonperipheral “washout”; (i) restricted diffusion; (j) Enhancement pattern; (k) Arterial peritumoral enhancement; (l) Intratumoral necrosis; (m) MRI liver cirrhosis; (n) Splenomegaly; (o) Ascites; Features (a), (e) to (i) were defined according to Liver Imaging Reporting and Data System (LI-RADS) v2018 [22, 23], while features (b) to (d) and (j) to (o) were non-LI-RADS definitions [24, 25]. The definitions of these features are detailed in Supplementary Tables S1 and S2.

### CODEX immune model construction

**Fig. 2:**
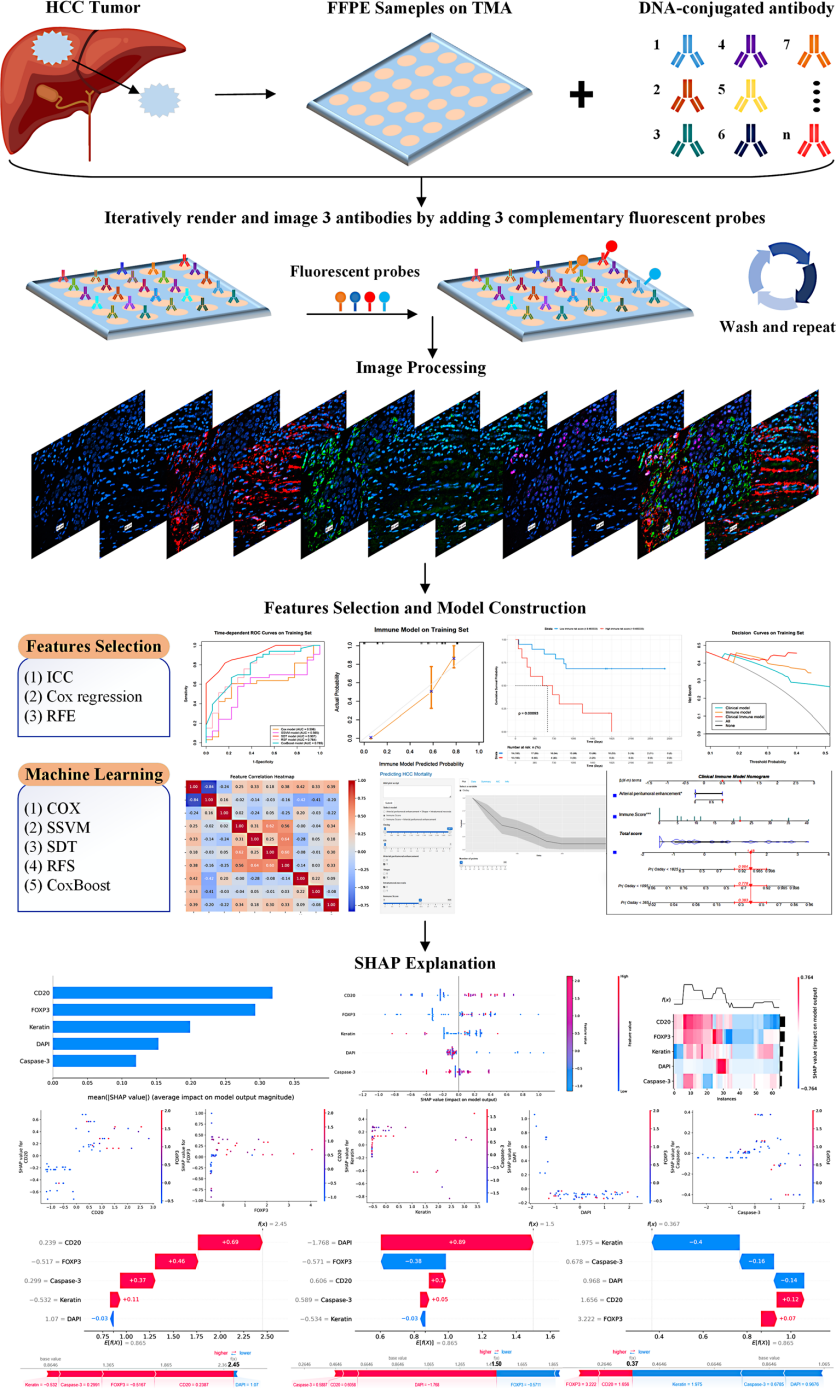
Workflow of CODEX and construction of the immune model

We selected 36 immune-related biomarkers for CODEX staining, including CD3, CD68, FOXP3, p-mTOR, Twist1, CD4, CD163, c-Myc, p-AMPK, Podoplanin, CD8, CD11C, HIF1a, CD107A, CD45RO, CD20, PD1, DAPI, Caspase-3, Vimentin, CD21, PD-L1, Keratin, Pan-CK, CD31, p-S6, αSMA, E-Cadherin, HLA-DR, CD44, CD45, p53, actin, Glypican3, and Hepar1. These biomarkers encompass a range of specificities, some being specific to certain cell types while some are expressed in multiple cell types. The workflow of the CODEX and the construction of the Immune model are illustrated in Fig. 2.

#### CODEX FFPE tissue staining and fixation

Tumor regions from Formalin-fixed, paraffin-embedded (FFPE) surgically resected HCC specimens (rather than biopsy samples) were selected to create a tissue microarray (TMA) with 1 mm diameter cores, which were then cut into 3 μm sections. The sections were mounted on coverslips, baked at 70℃ for 1 h, deparaffinized in xylene, rehydrated in ethanol, and washed in distilled water. After antigen retrieval, the coverslips were stained with a 36-marker antibody panel in 190 µl and incubated at room temperature for 3 h. CODEX multicycle imaging was performed using a Keyence BZ-X710 inverted fluorescence microscope with a CFI Plan Apo λ 20x/0.75 objective, a microfluidics instrument, and CODEX driver software (Akoya Bioscience). The final cycle included DAPI nuclear staining (Akoya Bioscience, #7000003).

#### Immune model construction

The raw image files were processed using CODEX software, creating seven-color overlay images in ImageJ to display selected markers. Protein expression within each tumor was analyzed using the CODEX Multiplex Analysis Viewer (Akoya Biosciences, version 1.5.0.8). All markers were normalized with the CODEX Analyzer. The 36 immune biomarkers and the number of detected cells were z-score normalized to maintain a consistent scale. Important immunomics features were selected using univariate Cox regression and the recursive feature elimination (RFE) method in training set, then included in five machine learning models: multivariate Cox regression, survival support vector machines (SSVM), survival decision tree (SDT), random survival forest (RSF), and CoxBoost. The best-performing machine learning algorithm was used to construct the immune model and calculate the immune score.

### Models Construction and Comparison

#### Clinical model construction

Perform univariate Cox regression analysis on all clinical, laboratory, and MR imaging variables, and include all features with *P* < 0.05 in the multivariate Cox regression analysis (forward LR) to select independent risk factors with *P* < 0.05 to construct the Clinical model.

#### Clinical immune model construction

The immune score obtained from the Immune model and the variables from the Clinical model were combined using multivariate Cox regression. This analysis aimed to identify independent risk factors for survival, forming the Clinical-Immune model.

#### Models comparison

The discrimination, calibration, and clinical utility of the three models were compared to select the best model for predicting 5-year survival. Discrimination performance was assessed using concordance indexes (C-indexes), time-dependent area under the curve (timeAUC), and time-dependent receiver operating characteristics (timeROC) curve analysis. Calibration curves were used to evaluate the consistency between predicted and actual outcomes. Decision curve analysis (DCA) was employed to assess whether the models provided clinical net benefit. The performance of the three models can be visualized using dynamic nomograms, which offer a convenient method for representing the predicted probabilities of survival at different time points.

### Statistical analysis

Statistical analyses were conducted using both Python (version 3.11.5) and R software (version 4.3.2).

Baseline data analysis: normality was assessed using histograms and the Shapiro-Wilk test. Homogeneity of variance was assessed using the Levene test. Data were considered normally distributed or with homogeneous variance when the p-value was greater than 0.05. For continuous variables following a normal distribution with homogeneous variance, data were presented as mean ± standard deviation and compared using Student’s t-tests. For variables not meeting these criteria, data were presented as median (interquartile range) and compared using the Mann-Whitney U test. Categorical variables were presented as case numbers (percentages) and compared using the chi-square test. A p-value less than 0.05 was considered statistically significant.

Model interpretation using the SHapley Additive exPlanations (SHAP) method involved both global and local strategies. For global interpretation, a summary bee swarm plot, heatmap of all patients’ SHAP values, and a bar graph of average SHAP values for each feature were used to provide an overview of feature importance and their impact on predictions across the dataset. These visualizations helped identify key features driving the model’s predictions. For local interpretation, waterfall plots and force plots were employed to explain predictions for individual patients. Waterfall plots showed how each feature contributed to a prediction, while force plots provided a detailed view of each feature’s impact on the prediction.

## Results

### Patient baseline characteristics

The baseline characteristics of the training and validation sets are summarized in Table 1. Among all 94 HCC patients, 46 were alive and 48 were deceased, resulting in a five-year survival rate of only 48.9%. In the training set (65 patients), there were 32 survivors with a median Osday of 1886.5 days, and 33 deceased patients with a median Osday of 1825 days. In the validation set (29 patients), there were 14 survivors with a median Osday of 1825 days, and 15 deceased patients with a median Osday of 545 days. The differences observed in both sets were statistically significant. Additionally, the variables indirect bilirubin (IBIL) and arterial peritumoral enhancement were also statistically significant in both sets.

### Clinical model

After performing univariate Cox regression analyses, only the variables carcinoembryonic antigen (CEA), total protein (TP), albumin (ALB), shape, arterial peritumoral enhancement, intratumoral necrosis, and MRI liver cirrhosis were included in the multivariate analysis. However, only shape (Hazard Ratio [HR]: 2.816, 95% Confidence Interval [CI]: 1.370–5.787), arterial peritumoral enhancement (HR: 2.599, 95% CI: 1.274–5.302), and intratumoral necrosis (HR: 3.037, 95% CI: 1.302–7.088) were independent risk factors, forming the Clinical model. For more details, refer to Table 2. The C-index and timeAUC of the model in the training set were 0.730 and 0.833, respectively, and in the validation set were 0.624 and 0.656, respectively.

**Table 2:** Variables for clinical model from univariate and multivariate cox regression analysis

Variable	Univariable Cox Regression				Clinical model			
	HR	lower 95%CI	upper 95%CI	*P*	HR	Lower 95%CI	Upper 95%CI	*P*
CEA	1.223	1.001	1.494	0.049				
TP	0.938	0.891	0.987	0.013				
ALB	0.925	0.857	1.000	0.049				
Shape	3.058	1.526	6.127	0.002	2.816	1.370	5.787	0.005
Arterial peritumoral enhancement	3.409	1.710	6.796	< 0.001	2.599	1.274	5.302	0.009
Intratumoral necrosis	2.966	1.283	6.857	0.011	3.037	1.302	7.088	0.010
MRI liver cirrhosis	2.517	1.037	6.109	0.041				

Abbreviations: CEA, carcinoembryonic antigen; TP, total protein; ALB, albumin; HR, Hazard Ratio; CI, Confidence Interval

### Immune model

**Fig. 3:**
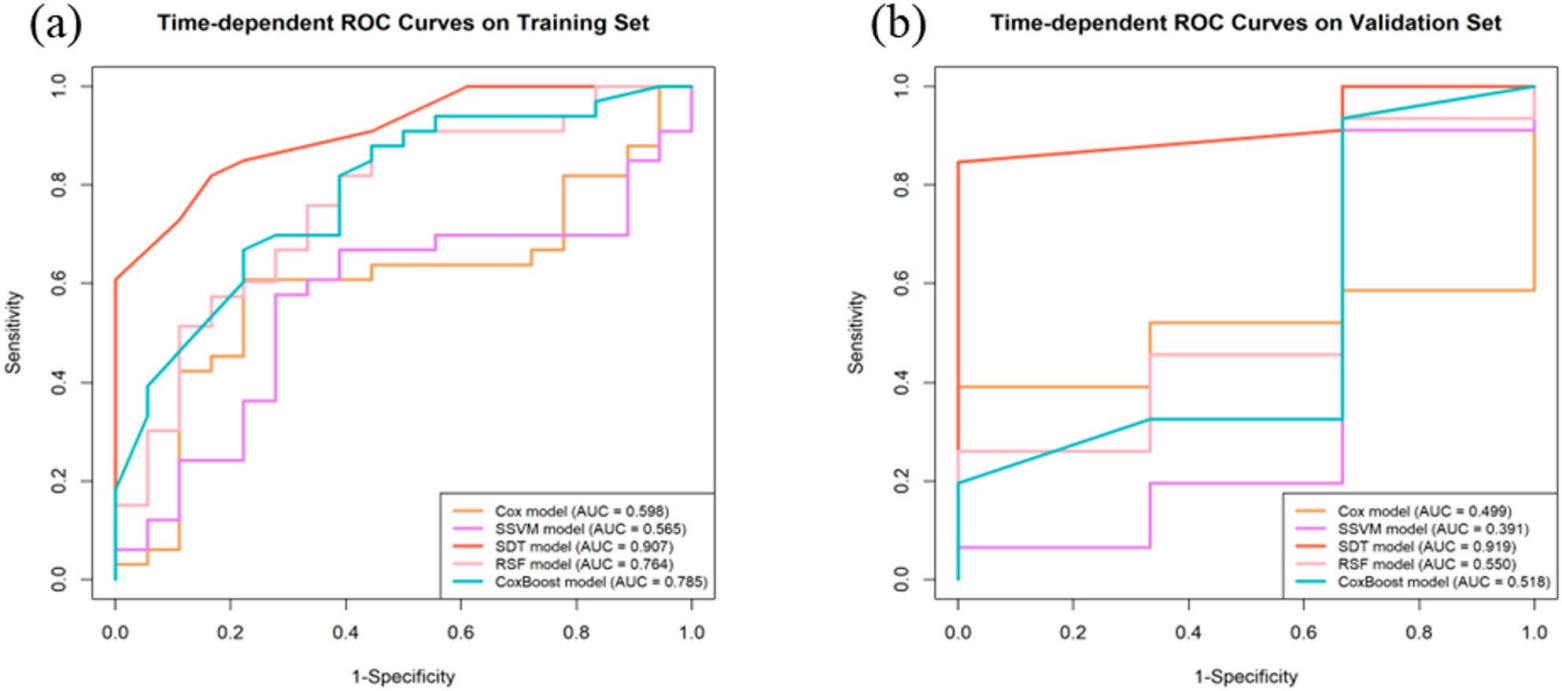
Time-dependent ROC curves of different machine learning models

Five important features (DAPI, FOXP3, Caspase-3, Keratin, CD20) were selected from the 36 CODEX immune features and used in five machine learning classifiers for modeling. Following comparison, the SDT classifier, which had the highest C-index and timeAUC in both sets compared to the other four machine learning models, was chosen as the final Immune model due to its superior performance and absence of overfitting. Specifically, its C-index and timeAUC were 0.832 and 0.907 in the training set, and 0.815 and 0.919 in the validation set. Table 3 shows the C-index and AUC values of these models, while Fig. 3 displays their timeROC curves on both sets.

**Table 3:** C-index and timeauc of different machine learning models

Machine Learning models		C-index	timeAUC
Cox	Training	0.666	0.598
	Validation	0.756	0.499
SSVM	Training	0.631	0.565
	Validation	0.797	0.391
SDT	Training	0.832	0.907
	Validation	0.815	0.919
RSF	Training	0.748	0.764
	Validation	0.711	0.550
CoxBoost	Training	0.762	0.785
	Validation	0.728	0.518

Abbreviations: C-index, Concordance index; timeAUC, time-dependent area under receiver operating characteristic curve; SSVM, Survival Support Vector Machine; SDT, Survival Decision Tree; RSF, Random Survival Forest

### Clinical immune model

The immune score from the Immune model and the three variables from the Clinical model underwent multivariate Cox regression analysis, resulting in the identification of two independent risk factors: immune score (HR: 1.105, 95% CI: 1.071–1.141) and arterial peritumoral enhancement (HR: 2.102, 95% CI: 1.029–4.297). These factors formed the Clinical Immune model. The model construction process and detailed results are presented in Table 4. The C-index and timeAUC were 0.852 and 0.969 in the training set, and 0.879 and 1.000 in the validation set.

**Table 4:** Variables for clinical immune model from multivariate cox regression analysis

Variable	Multivariable Cox Regression				Clinical Immune model			
	HR	lower 95%CI	upper 95%CI	*P*	HR	lower 95%CI	upper 95%CI	*P*
Immune Score	1.093	1.056	1.131	< 0.001	1.105	1.071	1.141	< 0.001
Shape	1.936	0.906	4.138	0.088				
Arterial peritumoral enhancement	2.084	1.027	4.232	0.043	2.102	1.029	4.297	0.042
Intratumoral necrosis	1.472	0.587	3.695	0.410				

Abbreviations: HR, Hazard Ratio; CI, Confidence Interval

### Models comparison and presentation

**Fig. 4:**
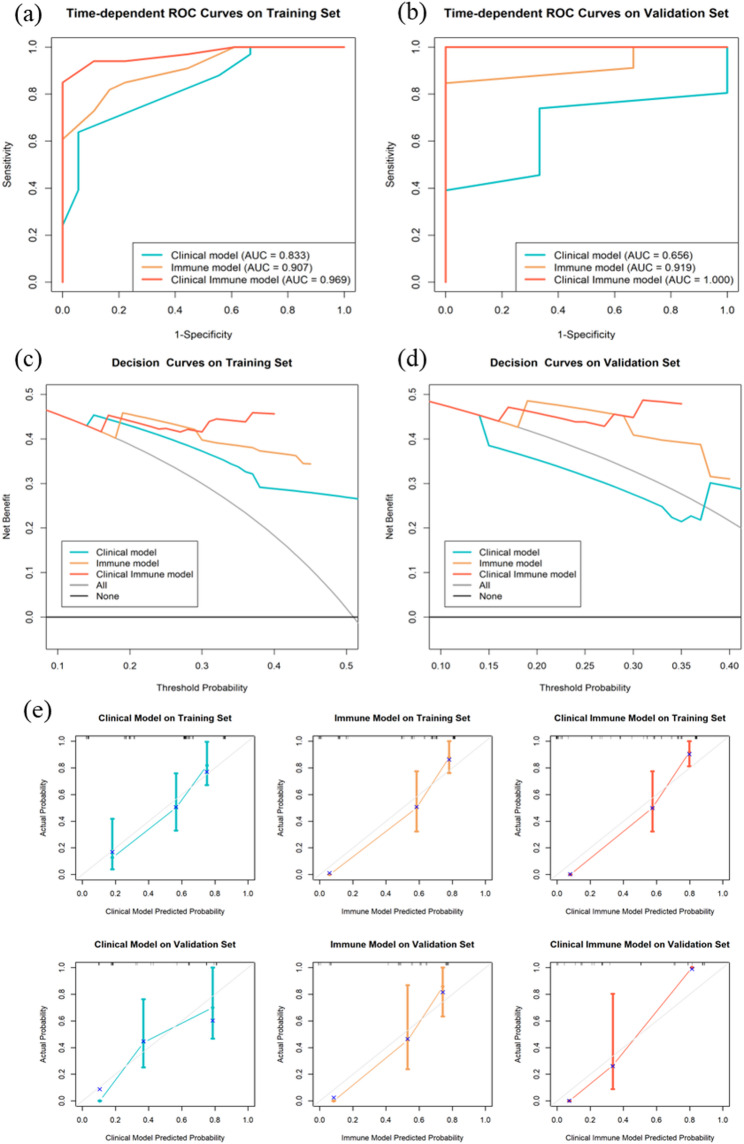
Three models for predicting HCC 5-year survival

**Fig. 5:**
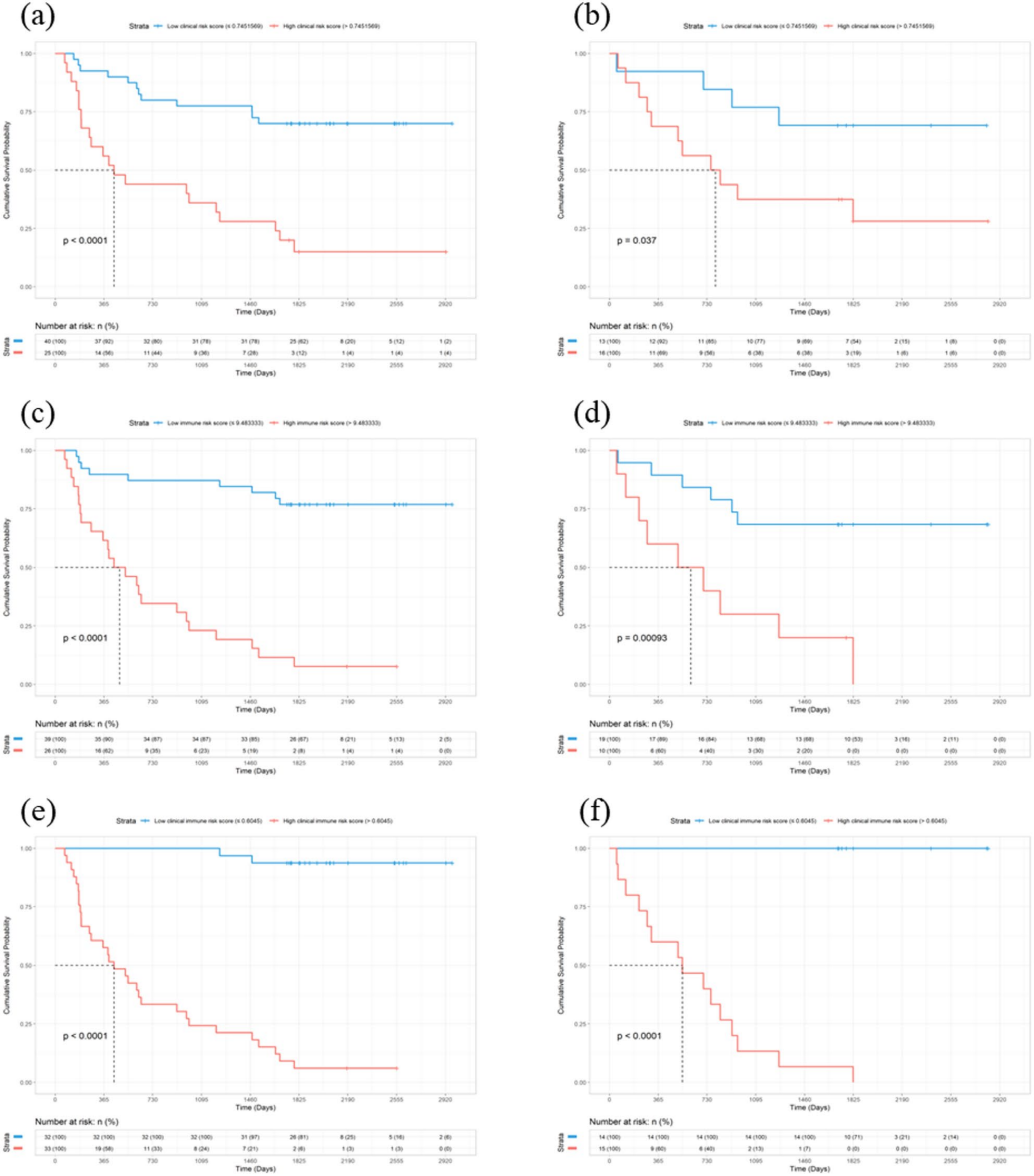
Kaplan-meier analysis of three models for predicting HCC 5-year survival**. ** (**a**), (**b**) Clinical model; (**c**), (**d**) Immune model; (**e**), (**f**) Clinical Immune model; (**a**), (**c**), (**e**) Training Set; (**b**), (**d**), (**f**) Validation Set

The C-index and timeAUC values of the Clinical Immune model were higher than the other two models in both sets (Table 5). Combined with the timeROC curves (Fig. 4a and b), this indicates superior predictive performance and better discrimination. Additionally, the DCA curves (Fig. 4c and d) and calibration curves (Fig. 4e) show that the Clinical Immune model achieves the highest clinical net benefit and that the predicted probabilities are closest to the actual probabilities. Furthermore, Kaplan-Meier (K-M) analysis comparing 5-year survival and death predictions of the three models (Fig. 5) shows that although all models can effectively distinguish 5-year survival, the Clinical Immune model performs the best. The K-M analysis of the three variables in the Clinical model is shown in Supplementary Figure S1.

**Table 5:** C-index and timeauc of three models for predicting HCC 5-Year survival

	Clinical model		Immune model		Clinical Immune model	
	Training	Validation	Training	Validation	Training	Validation
C-index	0.730	0.624	0.832	0.815	**0.852**	**0.879**
timeAUC	0.833	0.656	0.907	0.919	**0.969**	**1.000**

Abbreviations: C-index, Concordance index; timeAUC, time-dependent area under receiver operating characteristic curve

**Fig. 6:**
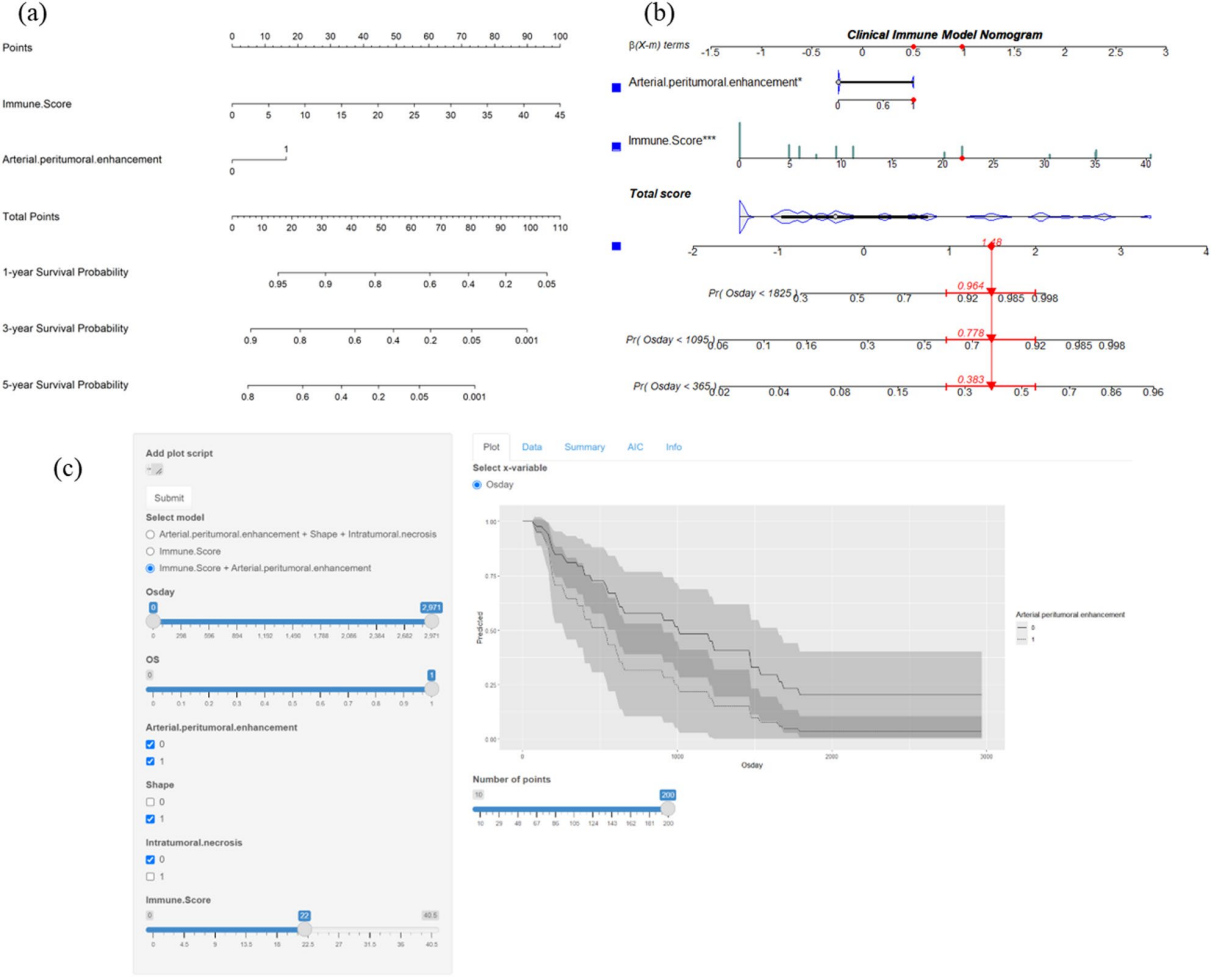
Clinical immune model utilization approaches. (**a**) Static Nomogram; (**b**) Dynamic Nomogram: Showing a 60-year-old male with an immune score of 22, arterial peritumoral enhancement, irregular shape, and no intratumoral necrosis. The nomogram predicts 1-year mortality rate of 0.383, 3-year mortality rate of 0.778, and 5-year mortality rate of 0.964. The patient ultimately survived for 1001 days. (**c**) Web App: Shows the same patient as in the dynamic nomogram, along with their potential survival curve

**Fig. 7:**
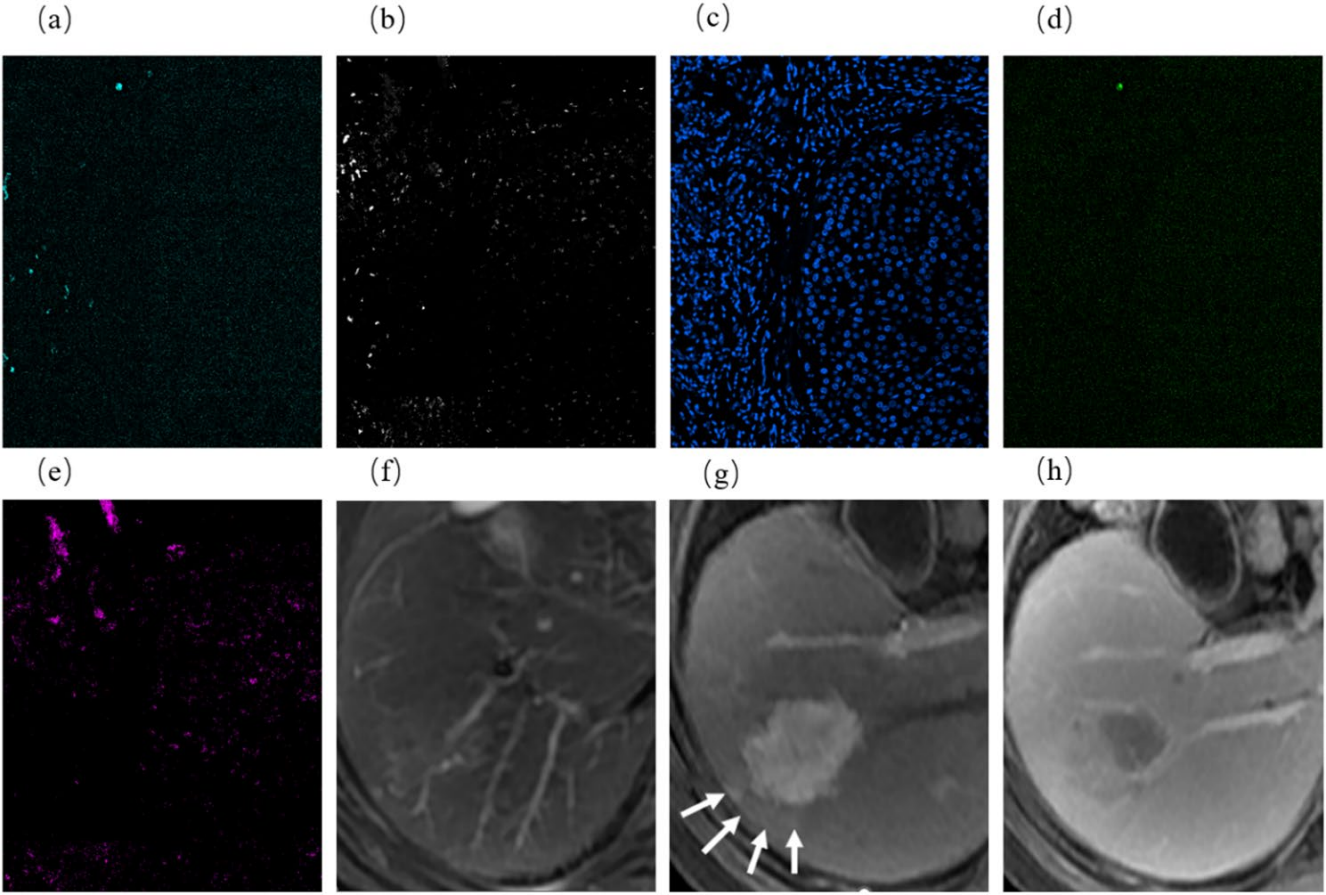
CODEX and MRI images of one patient. CODEX images: (**a**) CD20; (**b**) FOXP3; (**c**) DAPI; (**d**) Caspase-3; (**e**) Keratin. MRI: (**f**)T2WI (**g**) AP (**h**) DP. Showing a 60-year-old male with an immune score of 22, arterial peritumoral enhancement (**g**: white arrows), irregular shape, and no intratumoral necrosis. The patient ultimately survived for 1001 days

The Clinical Immune model is the final predictive model. To facilitate clinical use, we provide several methods for model utilization: static nomogram (Fig. 6a), dynamic nomogram (Fig. 6b), and a web app (Fig. 6c). The web app can be accessed at https://damiliu.shinyapps.io/Models/. This version allows for the simultaneous use of all three models, but Fig. 6c only shows the model we ultimately selected. Supplementary Figure S2 displays the presentation of the other two models. Additionally, Supplementary Figure S3 shows no collinearity among variables within the model. At the same time, we also provided CODEX and MRI images of this patient, as shown in Fig. 7.

### Model explanation

To understand how the Clinical Immune model’s two variables influence its predictions (see Table 4), we employed SHAP to interpret the immune score and the impact of key features on the SDT machine learning model. Detailed SHAP analysis elucidated the roles of five specific variables: DAPI, FOXP3, Caspase-3, Keratin, and CD20.

**Fig. 8:**
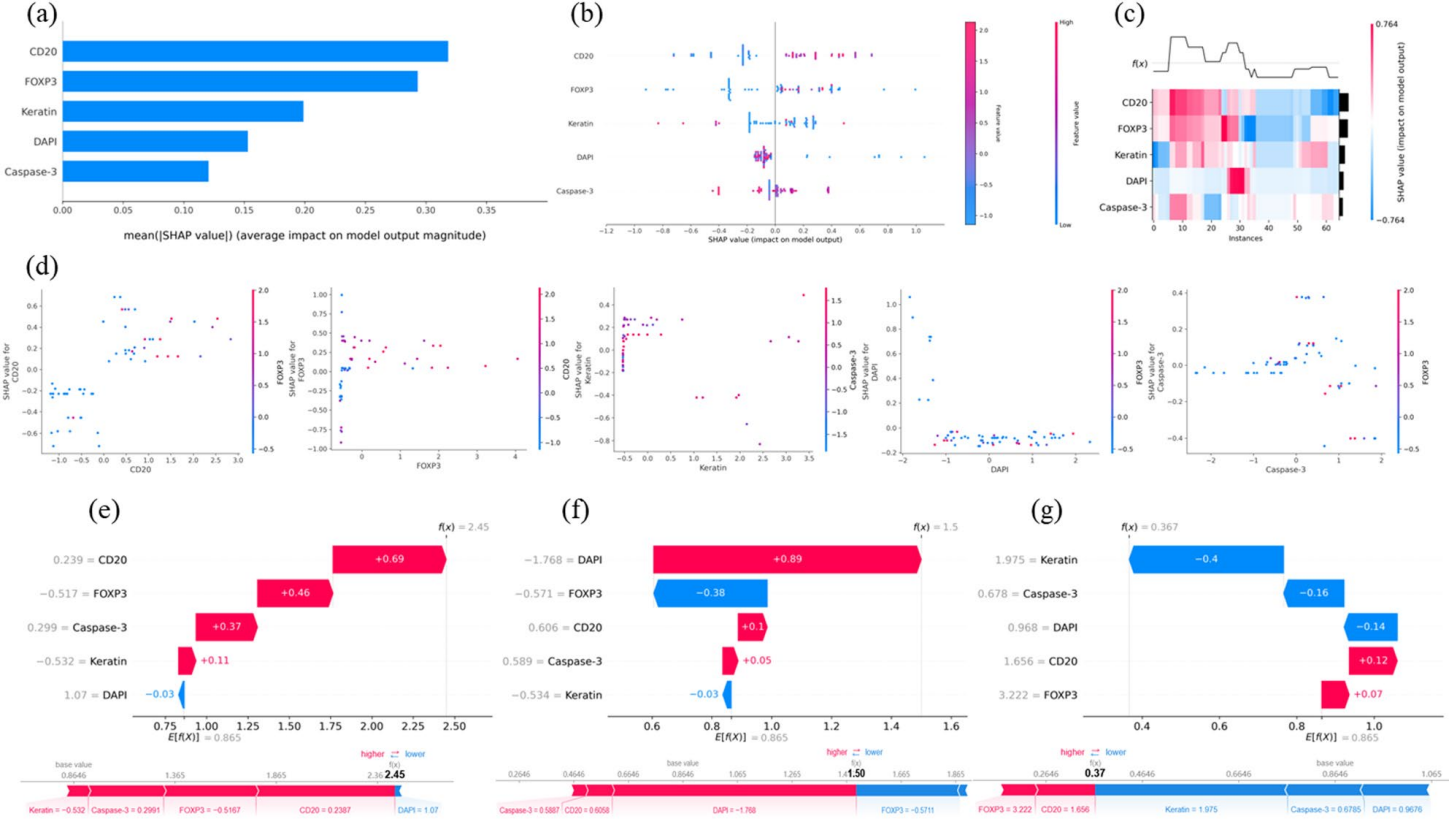
Clinical immune model SHAP explanation. (**a**) Feature Importance Bar Chart; (**b**) Summary Bee Swarm Plot: Each dot represents a single sample, with the color gradient indicating the feature value (from low to high). (**c**) Heatmap of SHAP Values: The horizontal axis represents individual samples, while the vertical axis lists the features. The color scale indicates the direction and magnitude of the feature’s impact, with red denoting a positive influence and blue indicating a negative one. (**d**) Scatter Plots of Individual Features: These depict the relationship between the SHAP values and feature values for CD20, FOXP3, Keratin, DAPI, and Caspase-3. (**e**) Waterfall Plot and Force Plot: For this patient, CD20, FOXP3, Caspase-3, and Keratin positively contribute to the model’s prediction, with SHAP values of 0.69, 0.46, 0.37, and 0.11, respectively, whereas DAPI exerts a negative effect with a SHAP value of -0.03. The total SHAP values sum up to 2.45 when added to the baseline value of 0.865, predicting the patient will die within 5 years. This patient had an immune score of 22 and ultimately survived for 1001 days. (**f**) Waterfall Plot and Force Plot: SHAP predicts this patient will die within 5 years; the patient had an immune score of 11.15 and ultimately survived for 1474 days. (**g**) Waterfall Plot and Force Plot: SHAP predicts this patient will survive beyond 5 years; the patient had an immune score of 7.5 and ultimately survived for 1904 days

The feature importance bar chart (Fig. 8a) displays the average impact of each feature on the model output, ranked by their mean SHAP values: CD20 > FOXP3 > Keratin > DAPI > Caspase-3. The SHAP summary bee swarm plot (Fig. 8b) illustrates the distribution of SHAP values for each feature across all samples, showing that high values of CD20 and FOXP3 are associated with a lower 5-year survival rate. The heatmap of SHAP values (Fig. 8c) provides a detailed view of the SHAP values for each sample and feature. Scatter plots of individual features (Fig. 8d) depict the relationship between the SHAP values and feature values for CD20, FOXP3, Keratin, DAPI, and Caspase-3, demonstrating how these feature values influence the model’s predictions to varying extents.

The SHAP waterfall plots and force plots (Figs. 8e-g) visualize the cumulative impact of features on the model’s prediction for three specific samples, showing how feature values and their corresponding SHAP values combine to form the final prediction output.

## Discussion

This study developed an interpretable machine learning model to predict postoperative survival in HCC patients by integrating CODEX immunomics and clinicoradiological features. Our findings indicate that the Clinical Immune model, which combines an immune score derived from CODEX and MRI features, significantly improves predictive accuracy and clinical utility compared to models based solely on clinical or immune features.

The Clinical model identified shape, arterial peritumoral enhancement, and intratumoral necrosis as independent risk factors for 5-year survival. These findings align with previous research highlighting the importance of radiological features in HCC prognosis [26–28]. Specifically, arterial peritumoral enhancement has been associated with poor outcomes due to its correlation with aggressive tumor behavior and microvascular invasion [28–30]. The immune model, which generates the immune score, incorporated five key immune features that significantly impact postoperative survival in HCC. DAPI is a nuclear stain that can indicate cell density and proliferation, with high expression potentially reflecting high tumor proliferative activity [31]. FOXP3 + regulatory T cells (Tregs) play an immunosuppressive role in the tumor microenvironment, inhibiting antitumor immune responses and promoting tumor evasion [32]. Caspase-3 is a key executor of apoptosis, and its activation level is related to the rate of cell apoptosis, reflecting the apoptotic dynamics of tumor cells [33]. Keratin is a major component of epithelial cells, and its abnormal expression is associated with tumor invasiveness [34]. CD20 + B cells have complex roles in the tumor microenvironment, potentially promoting immune responses or enhancing tumor progression by secreting immunosuppressive factors [35].

By combining the immune score and arterial peritumoral enhancement, the Clinical Immune model achieved superior predictive performance. This model provides a comprehensive assessment by integrating the tumor’s biological and radiological characteristics, thereby capturing the complexity of HCC more effectively than traditional models. It offers a robust tool for personalized prognosis in HCC patients, facilitating more informed clinical decision-making. The incorporation of SHAP enhances the model’s transparency, allowing clinicians to understand the contribution of individual features to the predicted outcomes [36]. This interpretability is crucial for gaining clinical trust and facilitating the integration of machine learning models into routine clinical practice. Furthermore, using CODEX technology to derive immune scores represents a significant advancement in assessing the tumor microenvironment. CODEX enables a detailed, multiplexed analysis of immune cell populations within the tumor, providing insights not achievable with traditional immunohistochemistry [37]. This comprehensive approach can potentially guide immunotherapy strategies by identifying patients who may benefit from targeted treatments based on their tumor microenvironment profile.

Consistent with transcriptomics-based classifications, the strong prognostic performance of our model supports the concept that HCC can be stratified into biologically distinct phenotypes with different clinical outcomes. In an integrative transcriptome meta-analysis, Hoshida et al. identified three robust molecular subclasses (S1–S3), in which S1/S2 are generally associated with more aggressive tumor biology and poorer prognosis, whereas S3 represents a more differentiated phenotype [38]. In our cohort, SHAP interpretation highlighted DAPI-derived nuclear density together with several immune/epithelial/apoptosis-related markers (including FOXP3, CD20, keratin, and caspase-3) as important contributors to risk estimation. These spatial proteomic features may represent phenotypic correlates of underlying molecular programs captured by transcriptomic subclasses. Although our approach does not replace transcriptomic profiling, CODEX-based spatial immunomics may provide complementary, interpretable information by preserving tissue architecture and offering spatial context at single-cell resolution, which may facilitate future clinical translation.

Regarding clinical relevance, we acknowledge that translating prognostic signatures into routine practice remains challenging, often due to cost and platform variability, the lack of spatial information in bulk profiling, and limited interpretability for bedside use. Our study aims to improve translational potential by integrating SHAP-based interpretability with spatially resolved immunomics: SHAP (e.g., waterfall plots) provides patient-level explanations that may enhance transparency and clinical confidence. Clinically, this tool is not intended for immediate indiscriminate deployment, but may support individualized surveillance intensity, facilitate risk communication, and enable the enrichment of high-risk patients for adjuvant strategies or clinical trials. Broader implementation of CODEX will require further standardization and feasibility evaluation; therefore, prospective multi-center validation is necessary before clinical use.

This study has several limitations. First, the retrospective nature of the study and the relatively small sample size may limit the generalizability of the findings. Larger, prospective studies are needed to validate the Clinical Immune model in diverse patient populations. Second, the selection of immune markers was based on available literature and our hypotheses; however, other relevant markers may have been overlooked. Future studies should explore a broader range of immune markers to further refine the immune score. Additionally, while the SHAP analysis provided valuable insights into the model’s predictions, it is inherently limited by the complexity of the underlying machine learning algorithms. Efforts should be made to improve the interpretability and usability of these models, ensuring they can be effectively utilized in clinical settings.

In conclusion, our study demonstrates that integrating CODEX immunomics and clinicoradiological features can significantly enhance the prediction of postoperative survival in HCC patients. The Clinical Immune model, supported by SHAP-based interpretability, offers a powerful tool for personalized prognosis and clinical decision-making in HCC management. Future research should focus on validating this model in larger cohorts and exploring additional immune markers to further improve its predictive performance.

## Supplementary Information

Below is the link to the electronic supplementary material.

Supplementary Material 1

Supplementary Material 2

## Data Availability

The original contributions presented in the study are included in the article/supplementary material. Further inquiries can be directed to the corresponding authors.
