# Supplementary material for: Developing an interpretable machine learning model via SHAP to predict HCC postoperative survival based on tumor immune microenvironment CODEX immunomics and MRI
Source: Cancer Imaging. 2026 Feb 14;26:42. doi: 10.1186/s40644-026-01006-y (PMC13011542; doi:10.1186/s40644-026-01006-y)
Supplement: Supplementary file 1 — Supplementary Material 1 [file 40644_2026_1006_MOESM1_ESM.docx]

**Table S1 Detailed MRI Scan Parameters**

| Sequences | Image plane | TR/TE (msec) | FOV (mm) | Flip angle | Thickness (mm) | Matrix | Scanning order |
| --- | --- | --- | --- | --- | --- | --- | --- |
| T2WI | A | 6000/85 | 420 × 420 | 160 | 8 | 512 × 512 | 2 |
| T1WI | A | 190/4.3 | 420 × 420 | 80 | 8 | 256 × 160 | 1 |
| DWI | A | 3650/75 | 420 × 420 | 90 | 8 | 200 × 200 | 3 |
| AP | A | 3.7/1.7 | 420 × 420 | 15 | 5 | 256 × 200 | 4 |
| PVP | A | 3.7/1.7 | 420× 420 | 15 | 5 | 256 × 200 | 5 |
| DP | A | 3.7/1.7 | 420 ×420 | 15 | 5 | 256 × 200 | 6 |

**Table S2 The definition of LI-RADS imaging Features**

| LI-RADS features | Definition |
| --- | --- |
| Tumor diameter | To measure the largest outer-edge-to-outer-edge dimension of an observation, follow these guidelines: (1) Include Capsule: Ensure the measurement includes any encapsulating capsule. (2) Choose Optimal Phase, Sequence, and Plane: Select the phase, sequence, and plane with the clearest margins. (3) Avoid Arterial Phase (AP) and diffusion-weighted imaging (DWI): Do not measure in the AP or DWI if margins are clear in another phase. AP measurements can overestimate due to peri-observation enhancement, and DWI measurements may be unreliable due to potential anatomical distortion. |
| Radiological capsule enhancement | Present Enhancing "Capsule": An enhancing capsule is characterized by a smooth, uniform, and sharp border that surrounds most (in the case of incomplete) or all (in the case of complete) of an observation. It is notably thicker or more prominent than the fibrotic tissue surrounding background nodules. This enhancing capsule is clearly visible as an enhancing rim in images acquired during the portal vein phase (PVP), delayed phase (DP), or transitional phase (TP).  Absent (Non-Enhancing "Capsule"): An absent or non-enhancing capsule lacks a visible capsule appearance as an enhancing rim. |
| Restricted diffusion | The intensity on DWI should be significantly higher than that of the liver, not solely due to T2 shine-through. Additionally, the apparent diffusion coefficient (ADC) should be markedly lower than that of the liver. |
| Nonrim APHE | This refers to unequivocal enhancement, either wholly or partially, in the AP, which is greater in attenuation or intensity than that observed in the liver. The enhancing part must exhibit higher attenuation or intensity than the liver in the AP. This should be contrasted with the concept of rim arterial phase hyperenhancement (rim APHE). |
| Rim APHE | This represents a spatially defined subtype of APHE, characterized by the most pronounced AP enhancement in the periphery of the observation. |
| Nonperipheral “washout” | This refers to a nonperipheral, visually assessed reduction in enhancement, either wholly or partially, relative to composite liver tissue from an earlier to a later phase. This results in hypoenhancement during the extracellular phase, occurring in the PVP or DP if an extracellular contrast agent or gadobenate is administered. With gadoxetate, this reduction in enhancement is seen during the PVP. |

**Table S3 The definition of Non-LI-RADS MRI features**

| Non-LI-RADS features | Definition |
| --- | --- |
| Tumor number | Only one lesion was solitary, and two or more lesions were multiple. |
| Shape | Tumors with a round or oval shape are categorized as regular, while those with other shapes, such as lobulated, star-like, or needle-like, are classified as irregular. |
| Margin | Nodular tumors with smooth boundary were smooth margin, non-nodular tumors with irregular contour and budding into the surrounding liver parenchyma were non-smooth margin. |
| Enhancement pattern | Typical enhancement meets the "wash in and wash out" enhancement, and the rest were typical; |
| Arterial Peritumoral enhancement | Defined as the enhancement outside the tumor boundary in the late stage of AP or early stage of PVP and extensive contact with the tumor edge, which becomes isointense during the DP. |
| Intratumoral necrosis | These areas typically appear as regions with no enhancement following contrast administration and may show high signal intensity on T2-weighted images (T2WI) and low signal intensity on T1-weighted images (T1WI). |
| MRI liver cirrhosis | Liver Morphology: Cirrhosis can lead to changes in the morphology of the liver, such as increased liver volume, irregular surface, or nodular appearance.  Signal Intensity: cirrhosis typically appears as signal abnormalities, where the signal intensity of liver tissue is altered compared to normal liver tissue. This may manifest as low signal on T1WI or high signal on T2WI. |
| Splenomegaly | Splenomegaly is characterized by an enlarged spleen, which can be visualized on MRI as an increase in spleen size compared to normal. This enlargement is typically assessed by measuring the craniocaudal length of the spleen. |
| Ascites | Ascites appears as areas of low signal intensity on T1WI and high signal intensity on T2WI, surrounding the liver and other abdominal organs. |

**
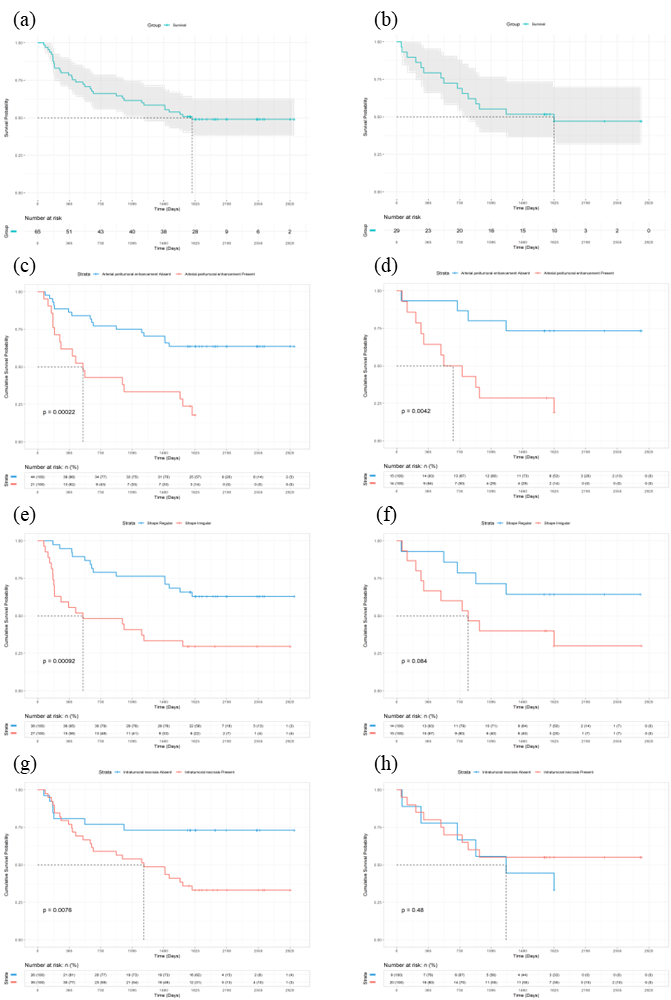
**

**Figure S1 Kaplan-Meier Analysis of Clinical Model Variables for Predicting HCC 5-year Survival**

(a), (b) Overall survival; (c), (d) Arterial peritumoral enhancement; (e), (f) Shape; (g), (h) Intratumoral necrosis; (a), (c), (e), (g) Training Set; (b), (d), (f), (h) Validation Set.

**
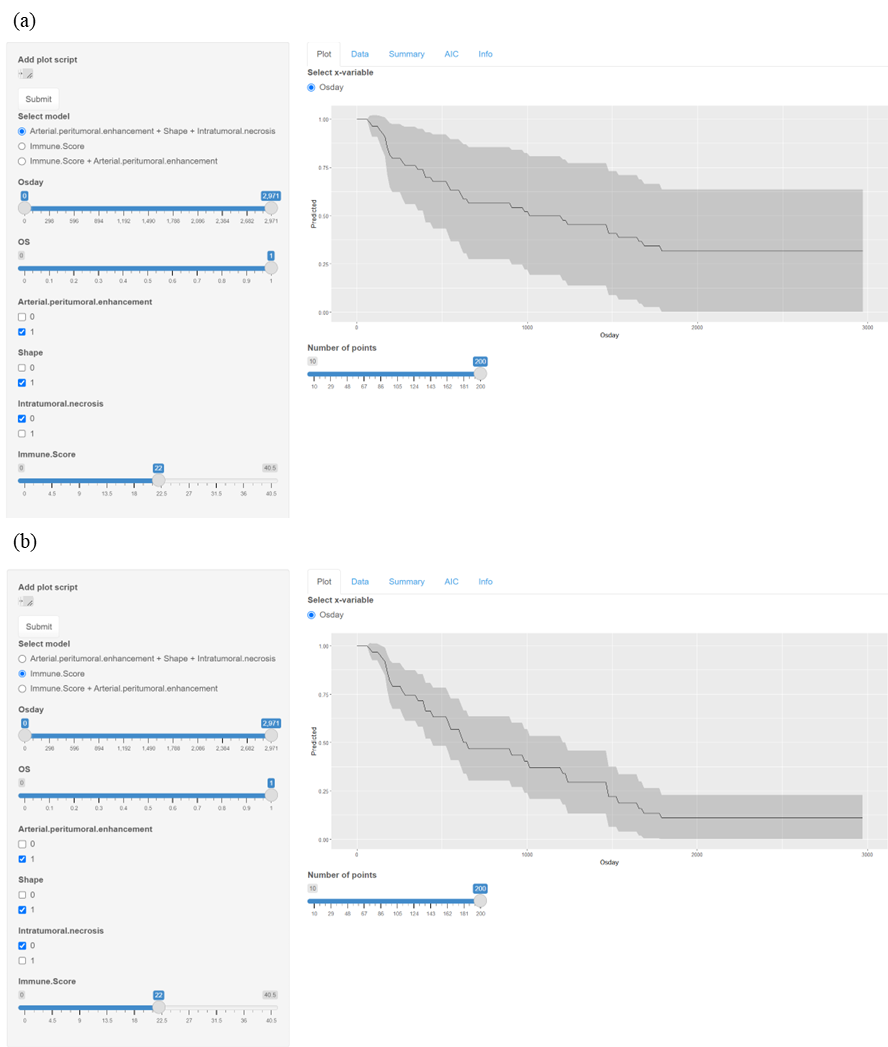
Figure S2 Web App of Clinical model and Immune model**

(a) Clinical model Web App showing the potential survival curve: A 60-year-old male with an immune score of 22, arterial peritumoral enhancement, irregular shape, and no intratumoral necrosis. The patient ultimately survived for 1001 days. (c) Immune model Web App showing the potential survival curve: Showing the same patient as in the Clinical model Web App.


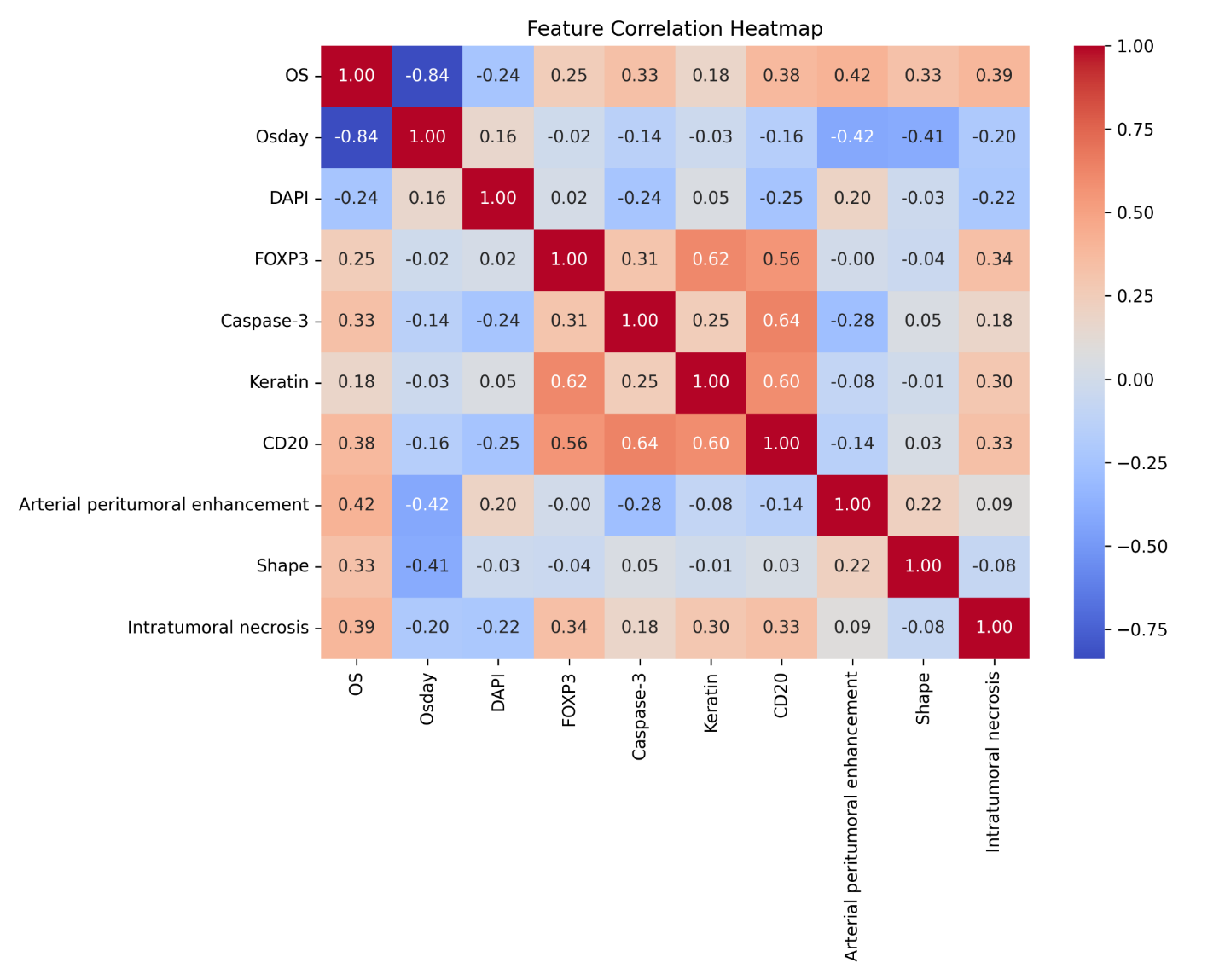
**Figure S3 Variable Correlation Heatmap**
