# Supplementary material for: Developing an interpretable machine learning model via SHAP to predict HCC postoperative survival based on tumor immune microenvironment CODEX immunomics and MRI
Source: Cancer Imaging. 2026 Feb 14;26:42. doi: 10.1186/s40644-026-01006-y (PMC13011542; doi:10.1186/s40644-026-01006-y)
Supplement: Supplementary file 2 — Supplementary Material 2 [file 40644_2026_1006_MOESM2_ESM.zip › 代码/ModelInfo.html]

```
Updated:  2024-05-02 17:57:24
```
